# Element Levels in Feathers of Atlantic Puffins (*Fratercula arctica*) in Iceland: Establishing Background Levels in an Arctic Migratory Species

**DOI:** 10.3390/toxics13020103

**Published:** 2025-01-28

**Authors:** Joanna Burger, Erpur Snær Hansen, Kelly Ng, Michael Gochfeld

**Affiliations:** 1 Division of Life Sciences, Rutgers University, 604 Allison Road, Piscataway, NJ 08854, USA; kelng@dls.rutgers.edu; 2 Environmental and Occupational Health Sciences Institute (EOHSI), Piscataway, NJ 08854, USA; mg930@eohsi.rutgers.edu; 3 Ecology & Evolution Graduate Program, Rutgers University, New Brunswick, NJ 08903, USA; 4 Consortium for Risk Evaluation with Stakeholder Participation, Rutgers University, Piscataway, NJ 08854, USA; 5 South Iceland Nature Research Centre, Ægisgata 2, 900 Vestmannaeyjar, Iceland; erpur@nattsud.is

**Keywords:** Cd, Pb, Hg, Se, puffins, seabirds, body feathers, Atlantic Puffin, *Fratercula arcticafa*, subsistence

## Abstract

Examining contaminant concentrations in birds in Arctic environments is important for managing species for assessing long-term trends. Recent reports on mercury (Hg) concentrations in Arctic species of seabirds has identified a need for data from missing regions or species. We measured arsenic (As), cadmium (Cd), chromium (Cr), lead (Pb), manganese (Mn), Hg and selenium (Se) in the body feathers of Atlantic Puffin (*Fratercula arctica*) from four colonies in Iceland in 2011 and one in 2009. Puffins forage on small fish at an intermediate trophic concentration. We found that (1) concentrations examined in the colony in 2009 were lower than in 2011 for all metals except As and Hg, and (2) concentrations of Cd and Se varied significantly among colonies for feathers collected in 2011. Pb concentrations in Puffin feathers in one colony were 14-fold higher in 2009 than in 2011 (mean of 805 ng.g^−1^ vs. 58 ng.g^−1^). The highest mean Hg concentration in 2011 was 362 ng.g^−1^ and was 4880 ng.g^−1^ for Se. The concentrations of Hg in the Atlantic Puffins reported in this study were similar to, or lower than those reported for the same species elsewhere and for Tufted Puffin from the Pacific.

## 1. Introduction

Increasing human populations, concentrations of people along coasts, climate change, and other global changes have resulted in governments, non-governmental agencies, and the public seeking information on the degree of contamination in different nodes on food chains, and in different geographical and ecological regions. This information is essential to formulating and supporting policies and regulations to protect human health and the environment, and to provide information to a range of stakeholders and Native American tribes [1,2,3]. Contaminants can enter different food chains through natural erosion, volcanoes, and other biogeochemical processes, and from anthropogenic sources such as mining, industrial disposal and erosion. Some chemicals are atmospherically transported all over the world, including isolated regions such as the Arctic and Antarctic [3,4,5,6]. Once dispersed on land and water, they enter aquatic food chains as well as terrestrial food chains. Such chemicals are taken up by plants that are eaten by other low trophic level organisms, which in turn are eaten by higher trophic level organisms. Understanding the threat that animals face because of contaminants is thus an important scientific and societal goal.

Most birds acquire heavy metals and other elements through the food and water they consume, although these contaminants are also passed from females to their embryos and developing chicks [7,8]. Birds that are intermediate or top trophic level predators typically accumulate higher concentrations than species that are at lower trophic levels, and are thus more vulnerable to toxic effects [9,10]. Seabirds are globally recognized as vectors of mercury and other contaminants derived from marine environments [11]. Heavy metals and other contaminants are a threat to birds, especially seabirds, because reduced reproductive success has been linked to arsenic (As), lead (Pb), and mercury (Hg) in birds [12,13,14,15,16]. To develop regulations and management strategies to reduce such contamination requires data on contaminant concentrations in a wide range of avian species, as well as the determination of potential effects from laboratory or field studies [17,18,19,20].

Monitoring and assessing contaminants in the Arctic has been an on-going program because of possible effects on ecosystems and human health. Interest in Arctic environments is increasing because climate change is resulting in increasing concentrations of metals due to permafrost melting, glacier melting, and changes in biological processes [6,21,22], as well as direct anthropogenic effects (e.g., fish farming [23] and mining [24]). Recently there has been considerable interest in compiling information on concentrations of Hg and Hg/selenium (Se) ratios in Artic shorebirds and seabirds [25,26,27,28,29,30]. These papers provide lists of species, with lists of Hg concentrations. Burger and Gochfeld 2009 [31], reported concentrations of Hg and other elements in breeding Tufted Puffins (*Fratercula cirrhata*) from the Aleutian Islands. However, summaries of other metals in a range of species are notably lacking, particularly regarding some species from the western Atlantic, such as the Atlantic Puffin (*F. arctica*).

In this paper we examine the concentrations of several heavy metals and other elements in Atlantic Puffins from Iceland. We examined As, cadmium (Cd), chromium (Cr), Pb, manganese (Mn), mercury (Hg), and selenium. We tested the hypotheses that (1) element concentrations did not vary in Puffin body feathers collected in 2009 (pilot study) and in 2011 in one colony (Vestmannaeyjar), and (2) element concentrations in Puffin feathers did not vary among four different Icelandic islands in 2011. We examined (1) Cd, Pb, and Hg, because they are the main and abundant contaminants in oceanic waters and they are known to have effects in wildlife and humans [32,33,34,35,36,37]; (2) As, because it poses a health problem for wildlife in marine systems [38]; (3) Cr, because it sometimes poses a problem in terrestrial systems [39]; (4) Pb, because of its known human and wildlife health effects [40,41]; and (5) Se, because of its known effects on aquatic birds [42,43,44,45]. Concentrations of Hg in feathers accurately reflect blood Hg when feathers were formed, making feathers a good indicator of circulating Hg that can be transported to other tissues [46,47,48].

Atlantic Puffins nest in colonies either on onshore peninsulas or offshore islands, in numbers ranging to over a million nesting burrows [49]. In Iceland they forage preferentially on small fish, the lesser sandeel (*Ammodytes marinus*) and capelin (*Mallotus villosus*), carrying them back whole in the beak to the colony to feed young chicks [50,51]. In Iceland Puffins have been hunted for food using pole nets since the 1880’s [52]. Understanding the concentrations of metals in feathers as indicators of internal exposure is thus of interest as a bioindicator for human exposure, and as a baseline for future comparative studies.

## 2. Materials and Methods

### 2.1. Study Sites

**Figure 1:**
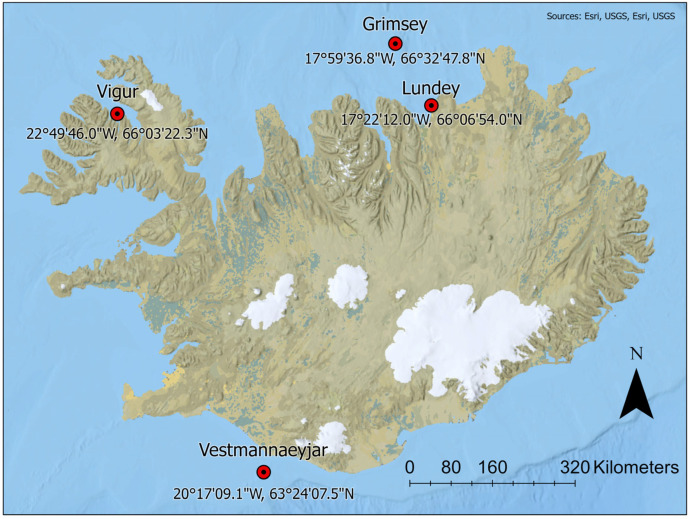
Locations of sampling sites for body feathers of Atlantic Puffin in Iceland in 2009 and 2011.

Body feathers were collected from the superlarge Westmans colony south of Iceland in 2009 (on sea) and 2011 (in colony), and at three additional colonies in 2011 representing the northwest, and northeast of Iceland (Table 1, Figure 1). At all colonies the main food is lesser sandeel and herring [53]. Iceland is located between boreal and sub-Arctic oceanic provinces; the southern Icelandic coast is dominated by saline, warm and nutrient-poor Atlantic seawater of tropical origin transported by the Irminger current. The north Icelandic coast is dominated by the East Icelandic current, an offshoot from the East Greenland current transporting fresh and cold polar water with much sea ice melt but is also highly variable annually due to the Irminger current [54].

### 2.2. Collecting Methods

Initially Atlantic Puffins were collected for diet investigation (27 April 2009, pre-breeding), and subsequently (July 2011, during the nestling period) puffins were collected by local hunters using pole nets (háfur) on 4 m long poles. The polenets were swung overhead as the birds flew by. Feathers were pulled from 4+ year birds, as determined by bill grooves [53,54]. Body feathers (about 40 per bird) were pulled from the breast and stored in a paper envelope to be sent directly to the Environmental and Occupational Health Sciences Institute (EOHSI) of Rutgers University in Piscataway, New Jersey. Sufficient feathers are pulled to allow some to be archived for future analysis, including for emerging chemicals. At EOHSI, feathers were kept at room temperature until analysis at EOHSI laboratories at Rutgers University. Metals in body feathers are accumulated during feather development, and when the feather is fully formed, there is no longer any blood remaining and the metals then remain constant, the feather serving as an archive of element concentrations at the time of molting [46,47,48]. Body feathers are small, and many are used in each analysis, eliminating part of the problem of using only one large wing feather (molted at one time). It should be noted that molting of body feathers in Atlantic Puffin occurs from December through March, with some continued shedding of body feathers in April [50].

### 2.3. Chemical Analysis

Feathers were analyzed as dry weight (in ng.g^−1^ = ppb). Sample sizes in 2011 were 15 to allow for individual variation. All laboratory equipment, utensils, and containers were washed in 10% HNO solution and rinsed with deionized water prior to each use. Care was taken to remove any non-feather debris before washing. Feathers were washed three times with acetone, alternating with deionized water, and were air dried. A 0.05 g (dry weight) sample of feather was digested in 4 mL 70% Fisher Chemical TraceMetal Grade nitric acid (Waltham, MA, USA) and 2 mL of deionized water in a microwave (MD 2000 CEM), using a digestion protocol of three stages of 10 min each under 50, 100, and 150 lb/in^2^ (3.5 u, and 10.6 kg.cm^2^) at 70% power. Subsequently, digested samples were diluted to 10 mL with deionized water.

Total Hg was analyzed by cold vapor atomic absorption spectrophotometry using a Perkin-Elmer FIMS-100 Hg analyzer (Waltham, MA, USA), of which about 85–90% is assumed to be MeHg [55]. Other elements were analyzed by flameless, graphite furnace atomic absorption. Instrument detection limits were 0.02 ng.g^−1^ for Cd, 0.15 ng.g^−1^ for Pb, 0.2 ng.g^−1^ for Hg, and 0.7 ng.g^−1^ for Se. All specimens were analyzed in batches with known standards, calibration standards, and spiked specimens. Blanks, standard calibration curves, and spiked matrix specimens were used to monitor assay performance for all batches. Certified reference material (CRM) DORM—“Dogfish Muscle. Certified Reference Material for Trace Elements” from the National Institute of Standards and Technology (NIST) was used for cold vapor atomic absorption spectroscopy (HG). “Trace Metals in Natural Water” from the National Institute of Standards and Technology (NIST) was used for Zeeman graphite furnace atomic absorption spectroscopy (As, Cd, Cr, Pb and Se) quality control. All concentrations are expressed in ng.g^−1^ (=ng/g = ppb) dry weight for feathers. Recoveries ranged from 86% to 101%. There were no batches with recoveries less than 85%. The coefficient of variation on replicate, spiked samples ranged up to 10%.

### 2.4. Statistical Analysis

Temporal and differences in element concentrations for Vestmannaeyjar were examined using Wilcoxon two-sample test, yielding an X^2^ statistic. Spatial comparisons among the 4 Puffin colonies were made using Kruskal–Wallis 1-way ANOVA, yielding an X^2^ statistic. A Conover–Iman test was used as a post hoc test to show the significant differences between colonies. Non-parametric statistics were used because they are more appropriate for small samples sizes. A probability of *p* < 0.05 was considered significant.

## 3. Results

Initially, body feathers were taken from Atlantic Puffins that were shot (2009), but in 2011 all feathers were collected from Puffins caught in pole nets. We observed significantly lower concentrations of five elements (Pb, Cd, Se, Cr, Mn) (Table 2). Mean Pb concentrations were 14-times higher in 2009; Cr concentrations were 4.4-times higher; Cd concentrations were 3-times higher; and Se concentrations were 1.6-times higher (Table 2). While Pb concentrations may partly reflect the method of collecting, this is not true for the other elements (see Section 4).

Feathers collected in 2011 showed few differences in element concentrations (Table 3). There were significant differences only for Cd and Se. Cd was higher in feathers of Puffins collected on Lundey and Vigur (the westernmost colonies) compared with the other two islands. In contrast, Se was lower in Puffin feathers collected from Vestmannaeyjar than those from the other, more northern colonies. Concentrations are rounded to three significant digits for As and Hg.

**Figure 2:**
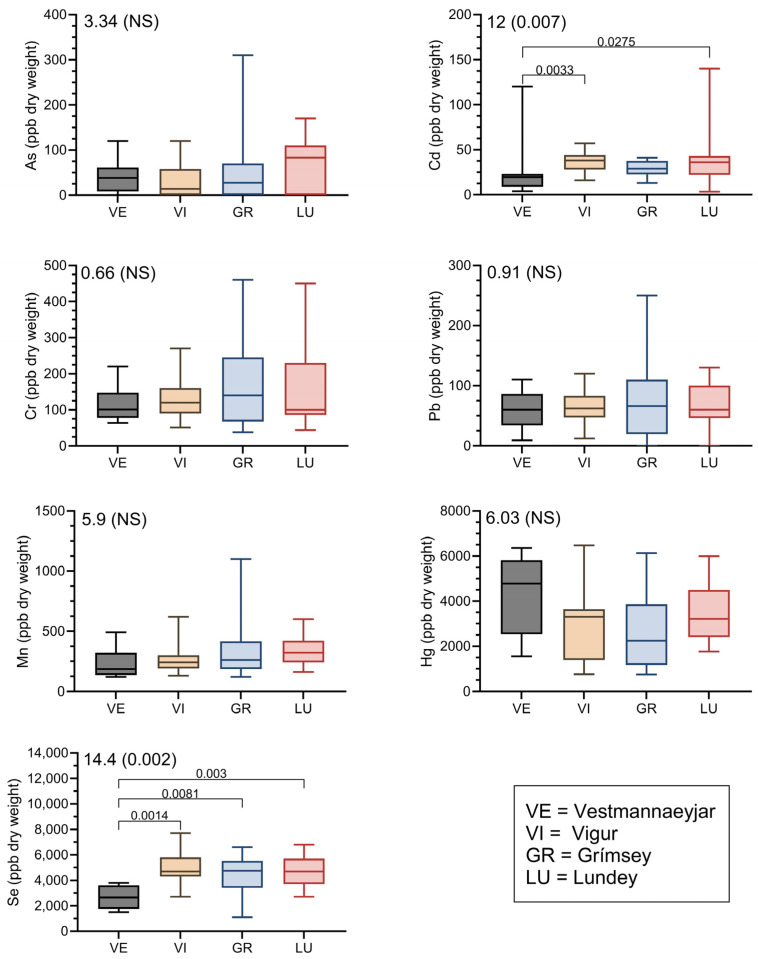
Graphical representation of metal concentrations in Atlantic Puffin from Iceland. Shown are medians (crossbar), 25th to 75th percentile (box), and highest and lowest values. Kruskal–Wallis chi-square values and *p*-values are shown in top left of each metal representation. Brackets in Cd and Se graphs show Conover–Iman post hoc test *p*-values.

There were differences in the profiles of metals in the Puffin feathers collected in 2011 (Figure 2). The range of element concentrations was greater on some islands, and for some metals, despite the same sample size being used for each metal. The graphs in Figure 2 show the median, 50% of samples (box) and the highest and lowest value; note the difference in scale among metals. For almost all metals, Grimsey Island had a greater range in metal concentrations. Hg and Se showed some of the highest relative variation.

## 4. Discussion

The results of this study on Atlantic Puffins from Iceland indicated that (1) several metals decreased from 2009 to 2011 in body feathers collected from Vestmannaeyjar, (2) there were few locational differences in metal concentrations for body feathers collected from four different nesting colonies in 2011 (only Cd and Se), and (3) there was much variation in metal concentrations in feathers from each colony. Each will be discussed below, as well as the implications; however, methodological issues are discussed first.

### 4.1. Methodological Issues

Potential methodological issues with element analysis in this study include method of collection, time of analysis, and variances within metals. In 2009, feathers were collected from Puffins that had been shot (with Pb shot), while all feathers collected in 2011 were collected from Puffins that were pole netted. Pb concentrations were significantly higher in Vestmannaeyjar Island Puffin feathers in 2009 compared with 2011 (the only island with a temporal comparison). The Pb shot used in 2009 might compromise Pb concentrations, but the feathers were washed three times, which should remove external contamination [56]. The method of killing would not have affected the other metals. At Vestmannaeyjar some metals were significantly higher in 2009 than in 2011. All feathers from both 2009 and 2011 were analyzed at the same time, using the same methods on the same equipment, eliminating differences due to time of analysis. Lastly, there were differences in the variances within metals (see below), yet the statistical tests still detected locational differences in the Cd and Se concentrations in feathers detected in 2011.

### 4.2. Temporal Differences in Metal Concentrations in Feathers from Vestmannaeyjar Island

Concentrations of Cd, Cr, Pb, and Mn (but not As and Hg) were significantly higher in Puffin body feathers in 2009 compared with 2011. These differences could be due to small sample sizes (10 vs. 15 Puffins for Vestmannaeyjar Island), use of Pb shot, yearly differences, and timing of collection (pre-breeding vs. mid-breeding). Because the sample sizes were not so different, and no clear differences in ranges occurred, this may not have been an issue. The use of Pb shot could account for the differences in lead concentrations, but the feathers were washed extensively, which typically removes external contamination. More importantly, the other metals that declined also declined drastically, which could not have been due to Pb shot. A third possibility is simply that there were significant differences in exposure (and accumulation) between the two years, which is difficult to conclude given the small sample sizes. Hg bioavailability in aquatic systems varies seasonally, as a function of geography, varying deposition, and methylation rates, not to mention the sizes of available prey [57,58]. There are significant differences in metal availability yearly, as well as seasonally [59].

The fourth possibility, that the timing of collection may be an important contributor, seems plausible to us, at least for some of the variability. The feathers collected in April 2009 were collected prior to the breeding season, while those collected July 2011 were collected in the mid-chick rearing phase when the Puffins have been on the colony breeding for weeks. Replacing body feathers occurs in July–September, after the breeding season but before migration [50], and thus reflects local exposures. Further, female birds sequester metals in their eggs as a method of ridding their body of contaminants [60]. There are two issues we did not explore but that require consideration: (1) the mobilization of contaminants from tissue stores changes internal Hg exposure (and thus could free Hg to be stored in feathers) [15], and (2) physiological differences and stress that occurs during the nesting season that can change physiology and metal dynamics that may be reflected in subsequent feather replacement [57,58]. Both are beyond the scope of this paper but deserve mention.

### 4.3. Differences in Element Concentrations Among Colony Sites in Iceland

One of our main objectives was to examine whether there were locational differences in element concentrations in four breeding colonies in Iceland. We drew two tentative conclusions from the study: (1) there were only two elements (Cd, Se) in which there were significant inter-colony differences, and (2) there was significant intra-colony variation in metal concentrations for most metals, in most colonies. In the case of the latter, inspection of Figure 2 shows Cd was the metal that showed the least variation (as defined by how clumped 50% of the values were). Vigur had relatively small variation for most metals except Hg. Hg showed some of the greatest variation in the Puffins from all four colonies. This indicates that future studies need a larger sample size; in our collective experience (JB unpubl. data), these variances are quite large. Given the importance of Hg in terms of potential health effects [34,35], this bears further investigation.

There were significant differences in concentrations of Cd and Se in the feathers of Puffins. Vestmannaeyjar had significantly lower Cd values, even though there was an outlier (refer to Figure 2). We have no explanation as to why it was lowest, although it also had the lowest concentration of Se, with little variation among values. The Vestmannaeyjar Puffin colony was the southernmost colony, and as such, may begin to suggest a south–north gradient, which also bears further examination.

### 4.4. Geographical Comparisons of Element Concentrations

Although there is voluminous information on contaminant concentrations in seabirds [10], there is comparatively little on Puffins, even for Hg [25,28]. In Albert et al.’s [25] recent review of Hg in Arctic seabirds, they mentioned Hg concentrations from only 11 Tufted Puffins from Aiktak Island in the Pacific and we examined body feathers from 39 Tufted Puffins from Amchitka and Kiska Islands (Alaska) in the Bering Sea (Table 4). Indeed, Pollet et al.’s 2022 review [28] reports Hg concentrations from Atlantic Puffin, which seems recent, but the data are from Thompson et al., 1992 [30] and report Hg concentrations as 1830 ng.g^−1^ in the pre-1930 period, and as 4030 ng.g^−1^ in the 1980–1990 period, and 4300 ng.g^−1^ in Atlantic Puffins in the early 1990s (mainly from the UK). In general, the Atlantic Puffins from Iceland had similar concentrations as Tufted Puffins from the Aleutian Islands [30] and Aiktak Island in the Pacific, where mean Hg concentrations were 3120 ng.g^−1^ [25].

### 4.5. Potential Effects of Hg

Heavy metals and other elements can have deleterious effects [10], and herein we consider only the Atlantic Puffin element concentrations from 2011. Hg is the element of greatest concern in marine environments [7], although more recently Se has risen to the fore because of its known mitigating effects on the toxicity of Hg (Ralston and Raymond 2018 [61,62,63]. Recently, Dietz et al. [64] conducted a risk assessment of the effects of mercury on North Atlantic wildlife and reported that some species (and sexes) showed mercury concentrations that were above the high-risk threshold. Moderate risk from Hg in feathers of seabirds is estimated to range between 7.92–23.8 μg/g (=7920–23,800 ng.g^−1^), and high risk to be above 23.8 μg/g [44,63]. In Atlantic Puffin from Iceland, Hg concentrations ranged as high as 6471 ng.g^−1^, near the moderate effects concentration, but the mean was well below effects concentrations. Pollet [28] reported the Hg average of the very few published Puffin reports as being about 4000 ng.g^−1^.

We also note that concentrations of Hg in tissues of birds and other biota are often used as bioindicators of potential human exposure. This is particularly true for Atlantic Puffins because they are eaten by some in Iceland, the Faeroes and some other areas of the Arctic and sub-Arctic [49,65]. Indeed, our samples were collected by hunters (through either being shot or captured in pole nets). There is a relationship between concentrations in blood, feathers and other tissues; this relationship needs to be determined for each species, along with information on human exposure amount and frequency. However, for birds in general, the concentrations of Hg in feathers far exceed the concentrations in muscle or other tissues [66]. The mean Hg value of 3230 ng.g^−1^ in feathers is below the toxic effects concentration for the birds themselves, and for top concentration predators that eat them (including humans). We suggest, however, that the muscle tissue of puffins should be examined directly for Hg concentrations to assess potential human exposure.

Se is both an essential element and toxic. Essential elements show a U-shaped dose–response curve with adverse effects at low concentrations (deficiency) and high concentrations (toxicity) [38], at least in mammals. Selenoenzymes are important parts of the body’s antioxidant protections; however, high Se concentrations can promote oxidative damage [45]. Sublethal Se effects in birds range from about 1.8 ng.g^−1^ to 27 ng.g^−1^ for lethal effects [10,62]. Thus, the Puffins from Iceland were in the potential sublethal effects range, but further investigation is required for puffins generally. While Se is toxic in its own right at high concentrations [44,62], it is also important to measure Se in connection with Hg because of the potential each has to mitigate the effects of the other [64]. Ralston and others [61] have proposed that a molar ratio of Se/Hg of 1 or above is protective against Hg toxicity, suggesting that Hg’s toxic effects are due to a disruption of Se biochemistry. However, this suggestion requires considerably more experimentation, including how any such ratio affects different organ systems.

At high levels, Pb, like other non-essential elements, can have an adverse effect on birds. In the Atlantic Puffins examined in this study, concentrations in 2011 ranged as high as 250 in ng.g^−1^ in 2011. However, this Pb concentration is well below the usually mentioned toxic concentration of 4000 ng.g^−1^ [40,41], and even in 2009, the concentrations averaged well below this concentration. The Atlantic Puffin population has declined substantially in Europe over the past decade. Breeding failures have been attributed to food shortages, particularly the intense interspecific competition for the declining population of Sand Eels, which has been accelerated by climate change. Human hunting contributes to the decline in Iceland and the Faeroes [67,68]. Based on our metals data from Iceland, showing concentrations below toxic effects concentrations, it appears that metals are not a significant contributor to mortality or population decline.

## 5. Conclusions

We reported element concentrations in body feathers of Atlantic Puffin from Iceland from one colony in 2009 and from four colonies in 2011, thus we had two years of data for one of the colonies. There were no significant locational differences, except for Cd and Se in 2011, and the differences were within an order of magnitude. Concentrations of most metals in Puffin feathers were lower in 2011 than in 2009 (except As and Hg). Although the feathers from 2009 were collected with lead shot (which could account for differences in lead concentrations), the differences in other elements were within an order of magnitude. The data presented provide a baseline for future studies, given the importance of climate change to element dynamics in Arctic environments, and they suggest that element contaminants are not a significant contributor to population declines.

## Figures and Tables

**Table 1:** Summary of locations where samples of Atlantic Puffin body feathers were collected. Colony burrow numbers are after Hansen et al. [49].

Location	Coordinates	Sample Size	Years Collected	Burrow Numbers
Grimsey I.	17°59′36.8″ W, 66°32′47.8″ N	15	2011	99,900
Lundey I.	17°22′12.05″ W, 66°6′50.41″ N	15	2011	36,500
Vestmannaeyjar	20°17′00.0″ W, 63°24′00.0″ N	15, 10	2009, 2011	1,102,000
Vigur I.	22°49′46.0″ W, 66°03′22.3″ N	15	2011	38,400

**Table 2:** Concentrations of elements in feathers of Atlantic Puffin from Vestmannaeyjar, Iceland in 2009 and 2011. Given are means ± SE and geometric means below. Comparisons are made with Wilcoxon two-sample test, yielding an X^2^ statistic. All values are in ng.g^−1^ (ppb dry weight). NS = not significant. Concentrations have been rounded.

Year	2009	2011	X^2^
N	15	10	
Pb	805 ± 202 595	58.4 ± 9.7 48	55 (0.0004)
Cd	78.2 ± 16.9 58.5	26.4 ± 10.6 17.3	75 (0.0062)
Se	4280 ± 317 4100	2670 ± 286 2520	76.5 (0.0071)
Cr	509 ± 111 387	117 ± 2.0 108	63 (0.0011)
Mn	361 ± 49.0 325	230 ± 40.7 204	89.5 (0.0357)
As	54.5 ± 13.3 29.4	41.3 ± 11.7 14.1	121.2 (NS)
Hg	3100 ± 408 2710	4250 ± 569 3830	158 (NS)

**Table 3:** Element concentrations in body feathers of Atlantic Puffin collected in Iceland in 2011. Given are means ± SE, with geometric means below means. Comparisons are made with Kruskal–Wallis 1-Way ANOVA, yielding an X^2^ statistic. Statistically significant differences are shown with *p*-values < 0.05. All values are in ng.g^−1^ (ppb dry weight). NS = not significant.

Location	Vestmannaeyjar	Vigur	Grimsey	Lundey	X^2^
N	10	15	15	15	
Pb	58.4 ± 9.7 48	65.3 ± 7.1 58.2	81.8 ± 17.2 41.0	71.2 ± 10.0 43.3	0.91 (NS)
Cd	26.4 ± 10.6 17.3	37.0 ± 2.7 35.5	29.3 ± 2.3 28.0	39.6 ± 8.0 31.1	12 (0.007)
Se	2670 ± 286 2520	4880 ± 335 4710	4420 ± 389 4070	4670 ± 311 4510	14.4 (0.002)
Cr	117 ± 16 108	133 ± 15.9 120	167 ± 33.4 128	165 ± 31.8 132	0.66 (NS)
As	41.3 ± 11.7 14.1	32.0 ± 9.8 3.7	53.1 ± 20.8 6.5	67.1 ± 13.9 13.5	3.34 (NS)
Hg	4250 ± 569 3830	3010 ± 412 2590	2610 ± 428 2130	3400 ± 326 3190	6.03 (NS)

**Table 4:** Comparison of Hg and other elements in body feathers of Atlantic Puffin from Iceland (2011, this paper) with those from Tufted Puffins from the Aleutian Islands, Bering Sea. Given are means + standard errors ng.g^−1^ for the 4 colonies in Iceland combined, and for the 2 Aleutian colonies. Kruskal–Wallis chi squares and *p*-values are given.

	Atlantic Puffin	Tufted Puffin	X^2^ (*p*)
Collection year	2011	2004	
Sample size	55	39	
Life cycle state	Breeding season	Breeding season	
			
Elements			
As	49 ± 8	136 ± 26	5.6 (0.02)
Cd	34 ± 3	80 ± 13	10 (0.002)
Cr	148 ± 14	1820 ± 230	68 (<0.0001)
Pb	70 ± 6	1260 ± 339	27 (<0.0001)
Mn	303 ± 24	622 ± 58	28 (<0.0001)
Hg ^a^	3230 ± 219	2540 ± 195	2.9 (NS)
Se	4290 ± 198	6600 ± 344	28 (<0.0001)

^a^ Hg in Tufted Puffins from Aiktak Island (Pacific [25]) has a mean concentration of 3120 ng.g^−1^ (*n* = 11), similar to that found in Iceland.

## Data Availability

The data are available from the corresponding author upon request.
